# Achievement of malaria pre-elimination in Cape Verde according to the data collected from 2010 to 2016

**DOI:** 10.1186/s12936-018-2376-4

**Published:** 2018-06-19

**Authors:** Adilson José DePina, El Hadji Amadou Niang, Alex Jailson Barbosa Andrade, Abdoulaye Kane Dia, Antonio Moreira, Ousmane Faye, Ibrahima Seck

**Affiliations:** 1 Ecole Doctorale des Sciences de la Vie, de la Santé et de l´Environnement (ED-SEV), Université Cheikh Anta Diop (UCAD) de Dakar, Dakar, Sénégal; 2 Programa de Pré-Eliminação do Paludismo, CCS-SIDA, Ministério da Saúde e da Segurança Social, Praia, Cape Verde; 3 Laboratoire d’Ecologie Vectorielle et Parasitaire, Faculté des Sciences et Techniques, Université Cheikh Anta Diop (UCAD) de Dakar, Dakar, Sénégal; 4 Aix Marseille Univ, IRD, AP-HM, MEPHI, IHU-Méditerranée Infection, Marseille, France; 5 Instituto Nacional de Gestão do Território, Praia, Cape Verde; 6 Programa Nacional de Luta contra o Paludismo, Ministério da Saúde e da Segurança Social, Praia, Cape Verde; 7 Institut de Santé et Développement, Université Cheikh Anta Diop (UCAD) de Dakar, Dakar, Sénégal

**Keywords:** Malaria, Elimination, Cape Verde

## Abstract

**Background:**

Malaria, despite being preventable and treatable, continues to be a major public health problem worldwide. The archipelago nation of Cape Verde is in a malaria pre-elimination phase with the highest potential to achieve the target goal of elimination in 2020.

**Methods:**

Nationwide malaria epidemiological data were obtained from the Cape Verde health information system that includes the individual malaria case notification system from all of the country’s health structures. Each case is reported to the surveillance service then to the National Malaria Control Programme, which allowed for compilation in the national malaria case database. The database was analysed to assess the origin of the malaria cases, and incidence was calculated from 2010 to 2016 by sex and age. The health centre, health district and month of diagnosis were evaluated, as well as the sex and the age of the patients, allowing a direct descriptive analysis of national data to provide an up-to-date malaria epidemiological profile of the country. Malaria cases were classified as imported or indigenous, and then, geographical analyses were performed using a unique Geographical National Code with Quantum Geographic Information System 2.16.2 software to map the cases by municipalities. The overall temporal evolution of cases was analysed to assess their monthly and yearly variations from 2010 to 2016.

**Results:**

Malaria is unstable in Cape Verde, with inter-annual variation and the majority of infections occurring in adult males (> 20 years). The indigenous cases are restricted to Santiago (96%) and Boavista (4%), while imported cases were recorded in all the nine inhabited islands, originating from neighbouring countries with ongoing malaria transmission; from Lusophone countries (25% from Angola, 25% from Guinea-Bissau), followed by the Republic of Senegal (12%) and Equatorial Guinea (10%). In 2010–2012, more imported (93 cases) than indigenous cases (26 cases) were observed; conversely, in 2013 and 2014, more indigenous cases (49) than imported cases (42) were reported. In 2015 there were 20 imported cases and only 7 indigenous cases. Finally, in 2016, there were 47 indigenous cases and 28 imported cases. The mapping of cases by municipality and country of origin was possible with GIS analyses.

**Conclusion:**

While Cape Verde remains on track to achieve malaria elimination by 2020 owing to the reduction of the annual incidence to below 0.1%, the country still records cases of indigenous and imported malaria. However, the indigenous cases are exclusively confined to the Santiago and Boavista islands, while the imported cases recorded nationwide originate only from the African continent, mainly from adult men from the Lusophone countries. Cape Verde needs to target interventions to remove residual foci on Santiago and Boavista islands to reduce malaria lethality to zero and prevent its reintroduction from African countries via transmission across the archipelago. Cape Verde is a good example of local authority’s commitment to tackle malaria and work towards its elimination by strengthening the health and surveillance systems.

## Background

Malaria continues to have a devastating impact on people’s health and livelihoods around the world, despite being preventable and treatable [1]. Globally, a 41% reduction in the incidence of malaria was recorded between 2000 and 2015. The risk of acquiring or dying from malaria has decreased by 37 and 60%, respectively, since 2000 [2]. Although significant progress has been made, an estimated 216 million new cases and 445,000 malaria-related deaths were recorded worldwide in 2016, mainly in sub-Saharan Africa. Indeed, the rate of malaria incidence and mortality decline has stalled and even reversed in some regions since 2014 [3]. Therefore, better characterization of malaria epidemiology in all countries with ongoing malaria transmission is a critical prerequisite to developing better-targeted control strategies [4]. The renewed engagement in malaria eradication efforts is perceptible in the global agenda [1, 5] with the launch of the WHO Global technical strategy for malaria 2016–2030 (GTS). Additionally, Target 3.3 of the Roll Back Malaria advocacy plan, Action and Investment to defeat Malaria 2016–2030 (AIM) and the Sustainable Development Goals (SDGs), is focused on AIDS, tuberculosis, malaria and neglected tropical diseases [4]. Malaria elimination is a national goal for many countries [6], and 40 of 91 countries and territories with ongoing malaria transmission are expected to reduce malaria incidence by 40% by 2020 [1, 2]. However, to achieve this goal, there is a need for political commitment at the highest level [1]. This suggests evidence-based re-orientation of malaria control programmes to target interventions where they are most needed and will have the expected impact. Furthermore, once the disease incidence is reduced to a level making its elimination possible, the identification of residual transmission foci and the correct and early treatment of all asymptomatic and symptomatic infections as well as better vector control targeting are critical to drive elimination and prevent post-elimination malaria resurgence [7].

**Fig. 1:**
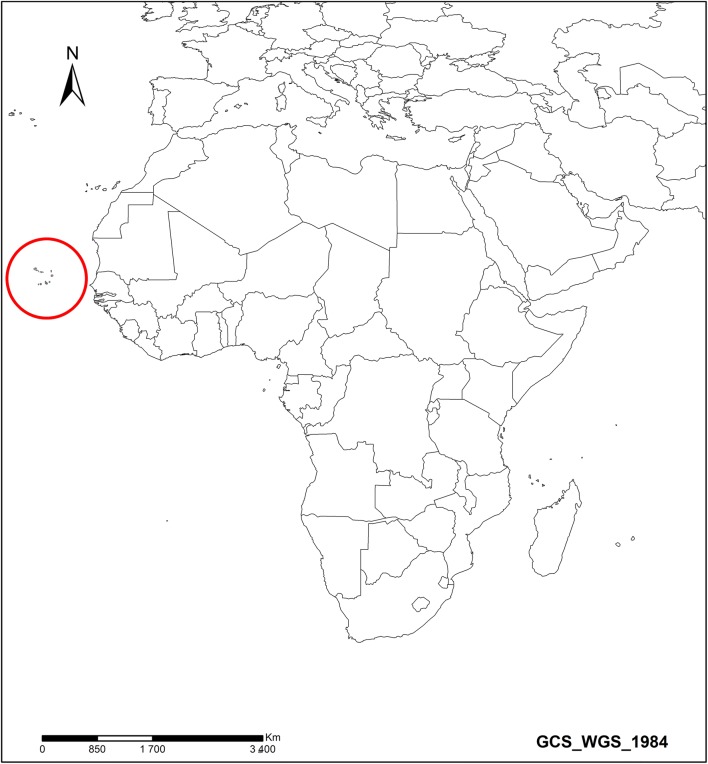
Geographical location of the archipelago of Cape Verde

Currently, globalization and transportation between areas of high to low endemicity enhance the risk of malaria resurgence in a post-elimination context and therefore jeopardize the elimination effort [8, 9]. Cape Verde is a volcanic archipelago located about 450 km from the West African coast, west of Dakar (Senegal), and occupies an area of 4033 km^2^. The archipelago consists of ten islands, of which nine are inhabited, and several islets (Fig. 1). With a total resident population of 537,661 inhabitants, the country has different malaria transmission levels for each island [10]. Malaria transmission is characterized by taking into account the presence or absence of the vector and the report of indigenous cases. Fifty-eight percent of the population of Cape Verde lives in high risk areas on islands where malaria vectors are present and where local transmission has been recorded previously. Thirty-seven percent of the population lives in intermediate transmission risk areas, which are characterized by the presence of the vector but no known local transmission. Finally, islands with neither the vector nor local transmission are classed as low risk zones and host 5% of the population [10, 11].

Since the first cases were recorded in the 16th century, during the colonization period of the islands [12–14], malaria has been shown to be relatively attenuated, but endemic, in the Cape Verde islands with recorded epidemics. More recently, the history of malaria in Cape Verde can be divided into several periods with different epidemiological profiles. In the 1940s, malaria was a serious public health problem with several major epidemics, reaching the status of hyper-endemicity with more than 10,000 cases and 200 deaths per year [10, 14]. A second period, which corresponds to the Global Malaria Eradication Programme between the 1950s and 1970s, introduced the implementation of a countrywide malaria control programme with semi-annual indoor residual spraying (IRS) campaigns with DDT. The IRS campaigns resulted in the interruption of disease transmission from 1967 to 1972 and then from 1983 to 1985, separated by periods of resurgence with hundreds of cases in subsequent years [10, 14]. Most recently, the country changed its national health policy in 2007, targeting the elimination of malaria by 2020 [15].

New strategies were adopted in 2017: a new treatment protocol, including the introduction of injectable primaquine; case surveillance with immediate responses to all cases by the health delegations; epidemiological and entomological research; the integrated fight of vectors with the fight against adult and larval mosquitoes with insecticides; environmental management and community sensitization.

All malaria-confirmed patients in Cape Verde are hospitalized and treated immediately with the first-line treatment, artemisinin-based combination therapy (ACT); in this case, artemether + lumefantrine were used for uncomplicated cases, and, since 2017, injectable artesunate has been for severe malaria cases. For all cases, primaquine is administered as a gametocytocidal on the first day of treatment to prevent human-to-mosquito transmission and interrupt the spread of the disease. For all patients hospitalized in the central or regional hospitals, a follow-up is done after their recovery by a delegation of the health system at days 04, 07, 14, 21, 28, 35 and 42 in line with the Malaria Treatment Protocol [16] to ensure correct case management. The post-treatment follow-up is done by RDT and microscopy for parasite detection and clinical evaluation.

This paper provides an epidemiological analysis of malaria in Cape Verde, summarizing Monitoring and Evaluation (M&E) activities, data collected from health facilities and reactive follow-up of detected cases from January 2010 to December 2016 as well as the geographical mapping of the reported domestic and international travel histories. Analysis of cases was conducted by sex and age, as well as classification by origin, and analysis of mortality data and the country’s challenges regarding the elimination of the disease are described.

## Methods

Data were collected from the health centres and the health delegations, which are the two services linked to each other to ensure uniformity and quality of the data disclosed in the Annual Statistical Reports of the Ministry of Health.

**Fig. 2:**
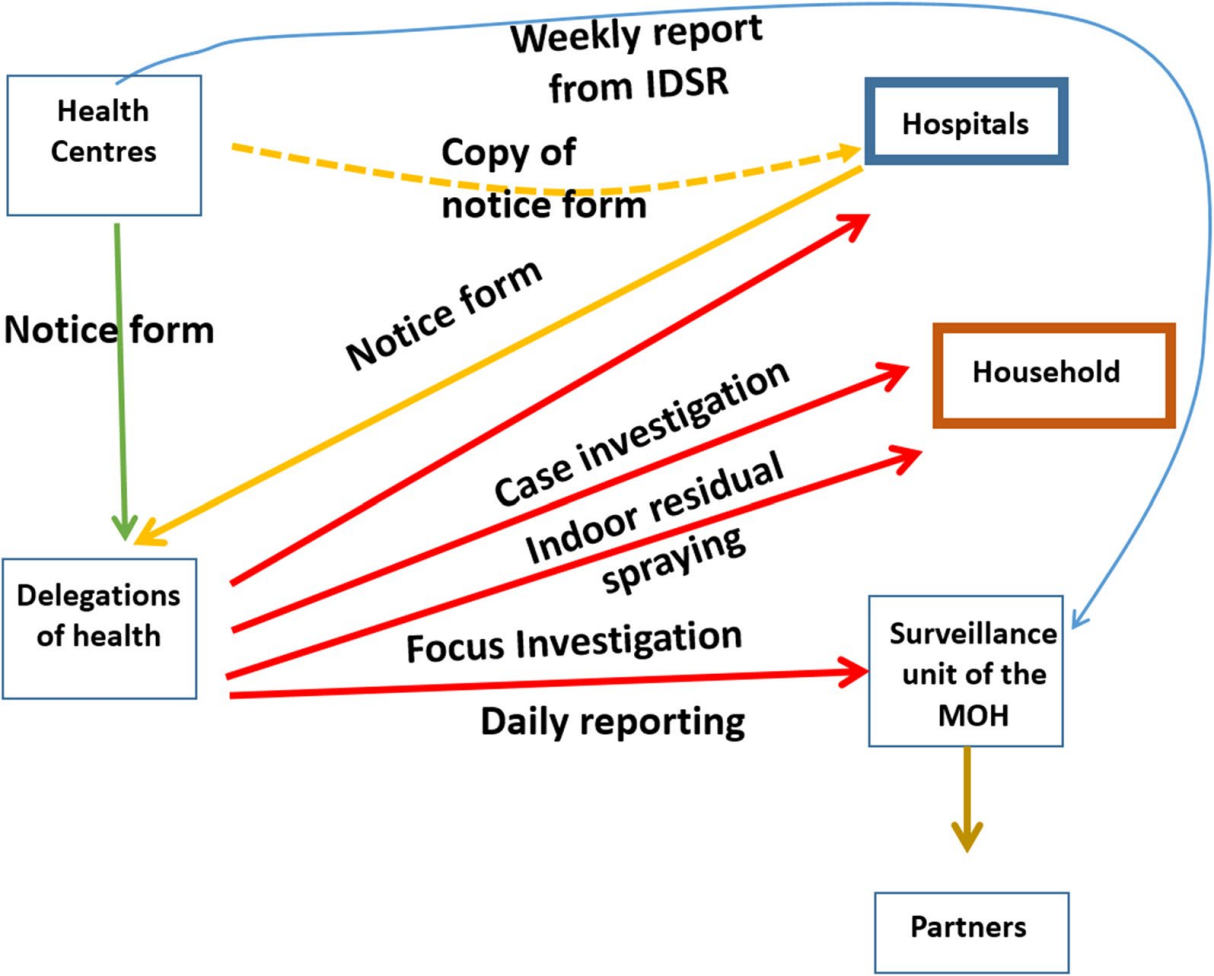
Cape Verde National Malaria Control Programme Surveillance System. Adapted by the authors

All malaria cases diagnosed in health facilities (hospitals, health centres, health posts, or in private services) were communicated immediately to the health delegation. All cases are classified in accordance with WHO guidance as imported, if the infection was acquired outside of the country, or as indigenous, if contracted locally with no evidence of importation. Health delegations immediately report any cases to the central level, the National Malaria Control Programme (NMCP) and the Integrated Surveillance and Response to Epidemics (SVIRE, in Portuguese), who then compile the data. Once a case is confirmed and notified, a follow-up is triggered. This consists of a visit to the patient’s home, reactive rapid diagnostic tests (RDT) in neighbouring houses, focal spraying activities, through IRS, in the case area and community awareness about the risk of malaria transmission (Fig. 2).

The investigation of each case is performed systematically at the level of the health delegation with the support of the central level. Notification is immediate with a unique form sent to the central level and included in the weekly notification form for diseases with epidemic potential. At the central level, data are analysed and disseminated to national authorities and other partners.

The mapping of cumulative malaria cases from 2010 to 2016 in Cape Verde was performed by pooling all cases notified from all health organizations. The incidence rate was calculated as [number of positive species − specific clinical cases notified/total population of the country] × 1000, and the lethality rate (%) was calculated as the [total of death by malaria/total of positive species − specific clinical cases notified] ×100. The age rate was grouped in accordance with WHO age standardization until 20 + , the same age groups used annually in the Annual Statistical Report of the Ministry of Health. The data were recorded in a Microsoft Excel sheet and structured by islands and their respective municipalities, with a unique National Geographical Code (NGC) used to link the database with the geographical data of the National Administrative Division retrieved from the National Institute of Land Management (INGT). The same procedure was applied to map imported cases for the same period (2010–2016). All maps were drawn with the 2.16.2 version of the QuantumGIS software to categorize cases of malaria.

## Results

A total of 312 malaria cases were reported in Cape Verde between 2010 and 2016 and confirmed by RDT and/or microscopy. These cases included 129 indigenous and 183 imported cases (Table 1). The disease incidence varied between years, with the majority of the cases (24%) recorded in 2016, followed by 2010 and 2014 (15%), 2013 (14%), 2011 and 2012 (12%) and 2015 (9%). As shown in Table 1, approximately 13% of all cases were recorded as severe malaria. Overall, the annual incidence of the disease was less than 1 case per 1000 inhabitants, with a median of 0.09, a minimum of 0.05 and maximum of 0.14 recorded in 2015 and 2016.

**Table 1:** Number of reported malaria cases in Cape Verde (2010–2016)

	2010	2011	2012	2013	2014	2015	2016	Total	%
Imported	29	29	35	22	20	20	28	183	59
Indigenous	18	7	1	23	26	7	47	129	41
Total	47	36	36	45	46	27	75	312	
% by year	15	12	12	14	15	9	24		
Simple	45	28	27	38	40	25	70	273	88
Complicated	2	8	9	7	6	2	5	39	13
Total	47	36	36	45	46	27	75	312	
Incidence rate (per 1000 inhabitants)	0.10	0.07	0.07	0.09	0.09	0.05	0.14		
Death	1	3	1	0	1	0	1	7	
Lethality rate (%)	2.1	8.3	2.8	0.0	2.2	0.0	1.3		
Sex									
Male	37	28	30	30	39	22	55	241	77
Female	10	8	6	13	7	5	20	69	22
ND				2				2	1
Total	47	36	36	45	46	27	75	312	
Cases by age group									
0–4 Years	1	2	0	2	1	1	1	8	3
5–9 Years	1	3	0	2	1	0	5	12	4
10–14 Years	0	1	0	3	3	0	2	9	3
15–19 Years	0	2	2	5	5	0	5	19	6
20 Years +	44	27	34	29	34	26	62	256	82
ND	1	1	0	4	2			8	3
Total	47	36	36	45	46	27	75	312	
Total of cases by municipality									
Paul	0	1	0	0	0	0	0	1	0.3
São Vicente	0	1	8	3	3	4	1	20	6.4
Ribeira Brava	1	0	0	0	0	0	0	1	0.3
Sal	2	1	3	0	1	1	3	11	3.5
Boavista	2	2	1	1	3	4	0	13	4.2
Cidade Velha	2	0	0	0	0	0	1	3	1.0
Praia	36	15	17	38	33	15	63	217	69.6
São Domingos	0	1	1	0	0	1	0	3	1.0
São Miguel	0	2	0	0	0	0	0	2	0.6
Assomada	1	5	3	1	2	2	4	18	5.8
Tarrafal	2	2	0	1	0	0	2	7	2.2
Santa Cruz	0	4	1	3	1	0	0	9	2.9
São Filipe	1	2	2	1	0	0	1	7	2.2
Total	47	36	36	48	43	27	75	312	

In general, with a total of 7 deaths, malaria lethality in Cape Verde varied between years. No deaths were recorded in 2013 and 2015, while the mortality rates varied from 8.3 in 2011 to 2.8 in 2012 and 1.3 in 2016. More males (77%) were affected by the disease than females (22%), and the majority of infections occurred in individuals older than 20 years of age, with 82% of overall cases compared with all other age groups, in which less than 6% was observed. In a group more affected by malaria in the rest of Africa (age range 0–4), only 3% was recorded in Cape Verde, 4% in the 5–9-year-old group, 3% in the 10–14-year-old group and 6% in 15–19-year-old group. Age information was missing for only 3% of patients. The importance of malaria-related death in the general mortality in the country is low during the period. While the average general mortality rate in the population is 5.1 per 1000 individuals (between 4.8 in 2010 and 5.3 in 2016), the average malaria-related death rate is 0.002 per 1000 individuals (between 0.0 and 0.06, in 2010).

**Fig. 3:**
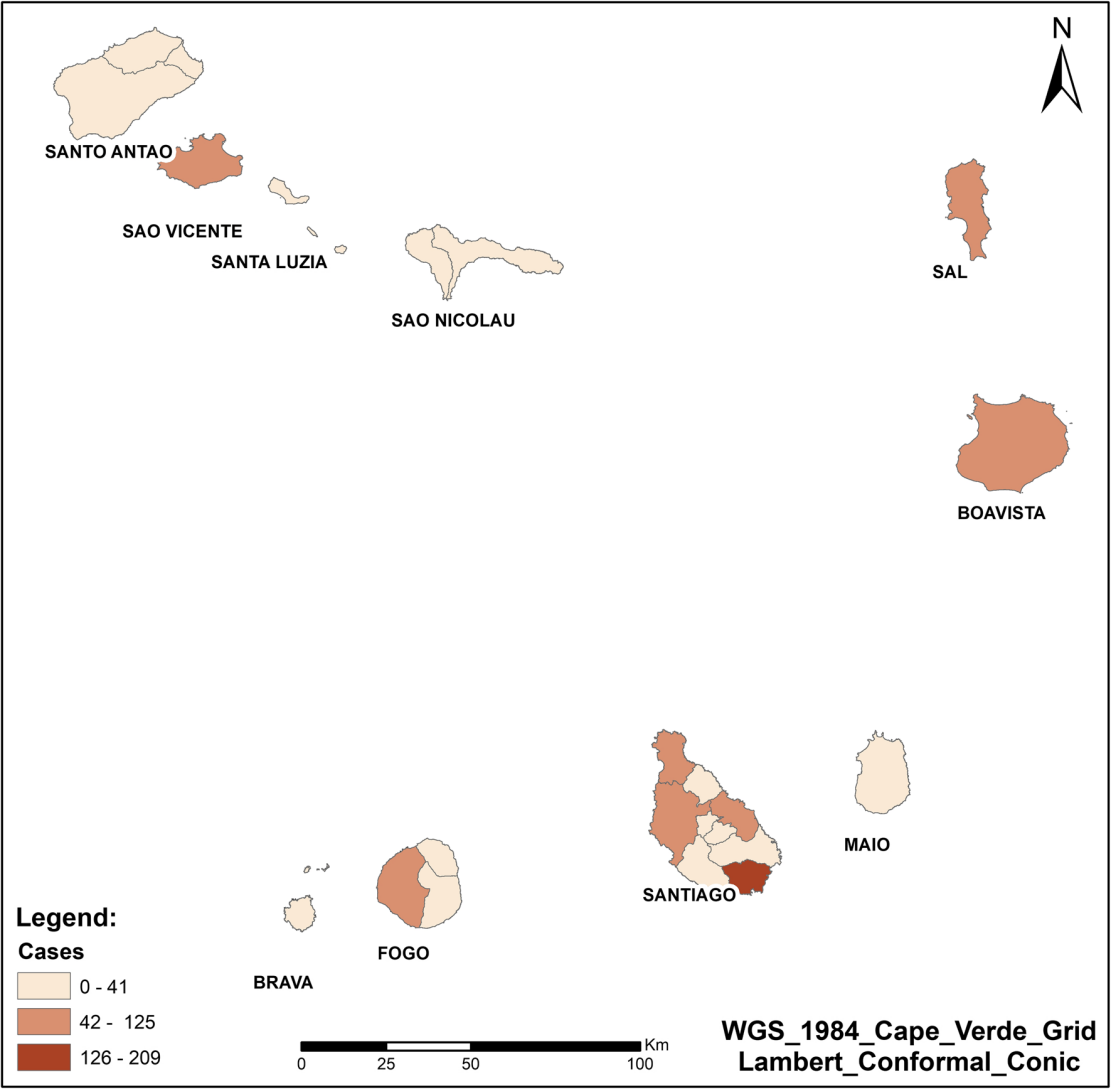
Number of malaria cases in Cape Verde, 2010–2016

During the study period, only 13 of 22 municipalities in Cape Verde reported indigenous or imported cases to the national level (Fig. 3). The majority of cases occurred in Praia (Santiago) (70% of cases), followed by the municipality of São Vicente (6.4%), Assomada (5.8%) (in Santiago), Boavista (4.2%) and Sal (3.5%) islands. All other remaining municipalities reported less than 3% of cases.

**Fig. 4:**
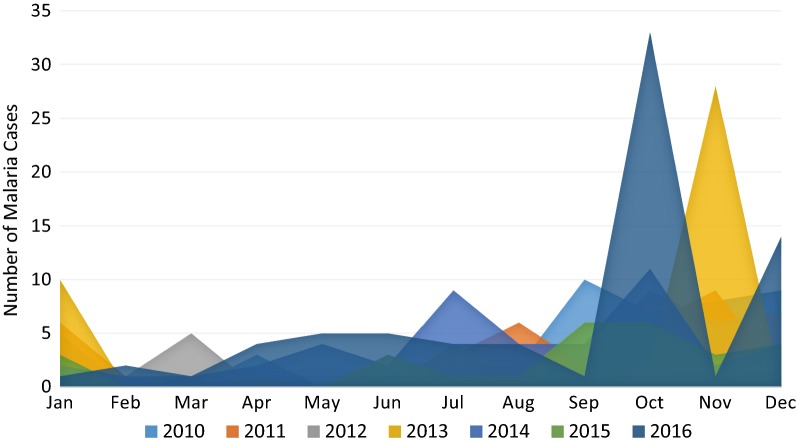
Monthly evolution of malaria incidence in Cape Verde, 2010–2016

From 2013 to 2016, the majority of cases were recorded during the last trimester of each year, varying monthly from 24% in October and 18% in November to 13% in December (Fig. 4). Approximately 9% of cases were reported in January, 2% of cases each in February and March, 3% in April and May, 4% of cases in June, 8% in July and September and 4% in August.

For each malaria case reported, imported or indigenous, a set of actions are carried out immediately. In particular, an epidemiological survey through questionnaires and reactive screening were undertaken in the household where the case was reported, focal treatment by IRS was performed in all houses within a 100 m radius of detected cases and the cases were followed-up after initial treatment. The majority of cases were detected passively; only 2 cases were identified during the reactive research in 2016, in the community of Fonton in Praia, which reported 23.4% of indigenous cases during the period (2010–2016). To combat the only vector species associated with transmission of the malaria parasite found in the country, *Anopheles arabiensis*, IRS with 5% deltamethrin was adopted as the standard strategies, and recent studies show the susceptibility of the species to this insecticide. IRS campaigns are carried out twice a year, especially in communities of municipalities considered to be at high risk for the disease, based on the presence or absence of vector and indigenous cases. LLINS is rarely used; there are no distribution campaigns, and it is only used in specific cases for migrants from endemic countries. After confirmation, patients are given an LLIN to prevent transmission. Antilarval control is another priority strategy in the country, which involves temephos larvicide, larvivorous fish and environmental management activities, especially in Praia, Assomada and other municipalities of Santiago, in recent years (after 2015).

### Indigenous malaria cases

**Fig. 5:**
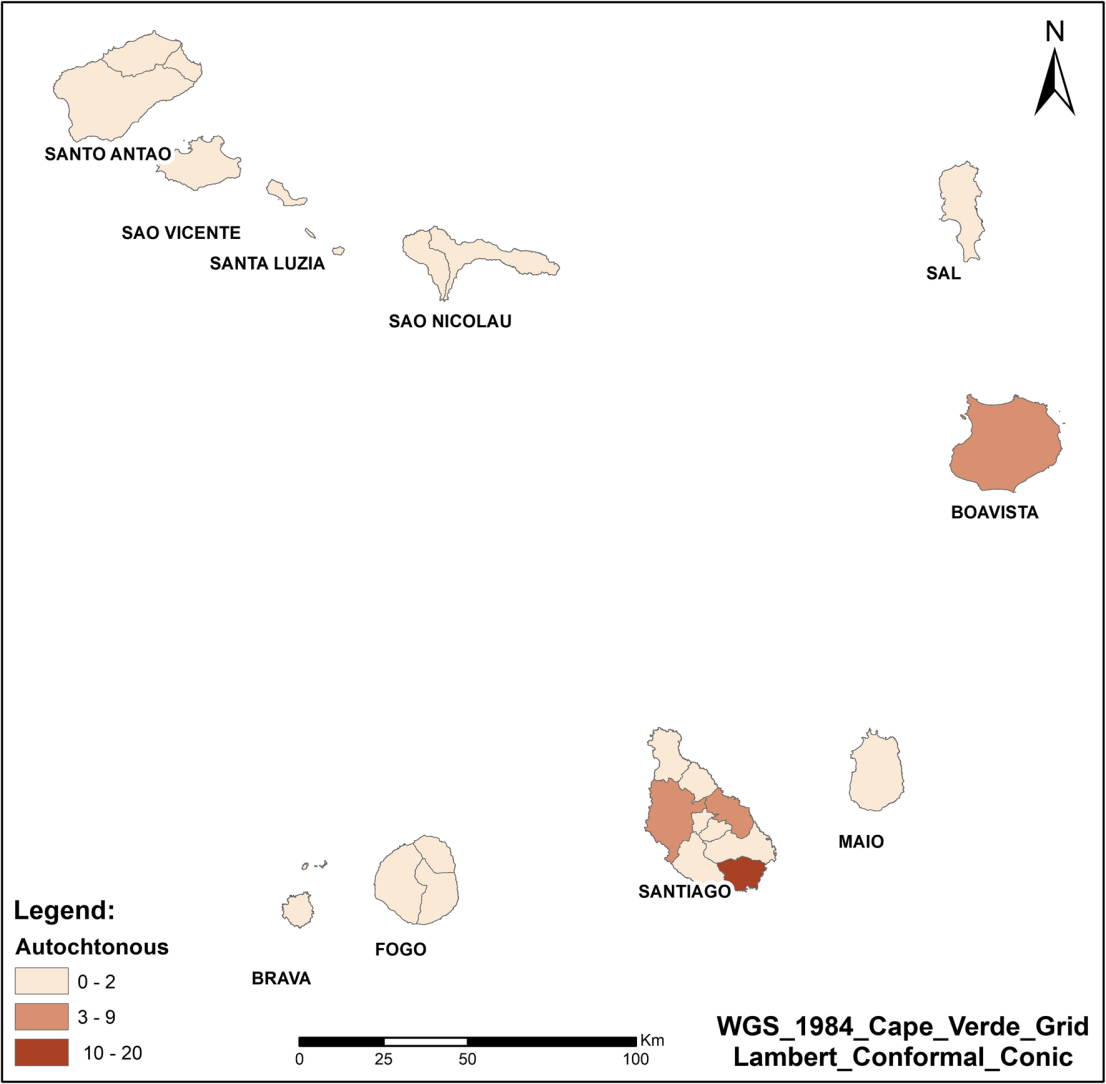
Number of indigenous cases of malaria in Cape Verde, 2010–2016

From January 2010 to December 2016, 41% (129/312 cases) of indigenous malaria cases were recorded. All of these cases were recorded in only two islands with the majority (96%) occurring in Santiago, and the remaining cases (4%) occurring in Boavista (Fig. 5).

The island of Santiago has the highest number of cases. Of the total cases reported in the country (indigenous and imported), 83% were on Santiago island. With regards to indigenous cases in Santiago, the majority are in the capital city, Praia (90%), followed by the municipalities of Santa Cruz (5%), Assomada (3%), São Miguel (3%), and Cidade Velha (1%).

### Imported cases

**Fig. 6:**
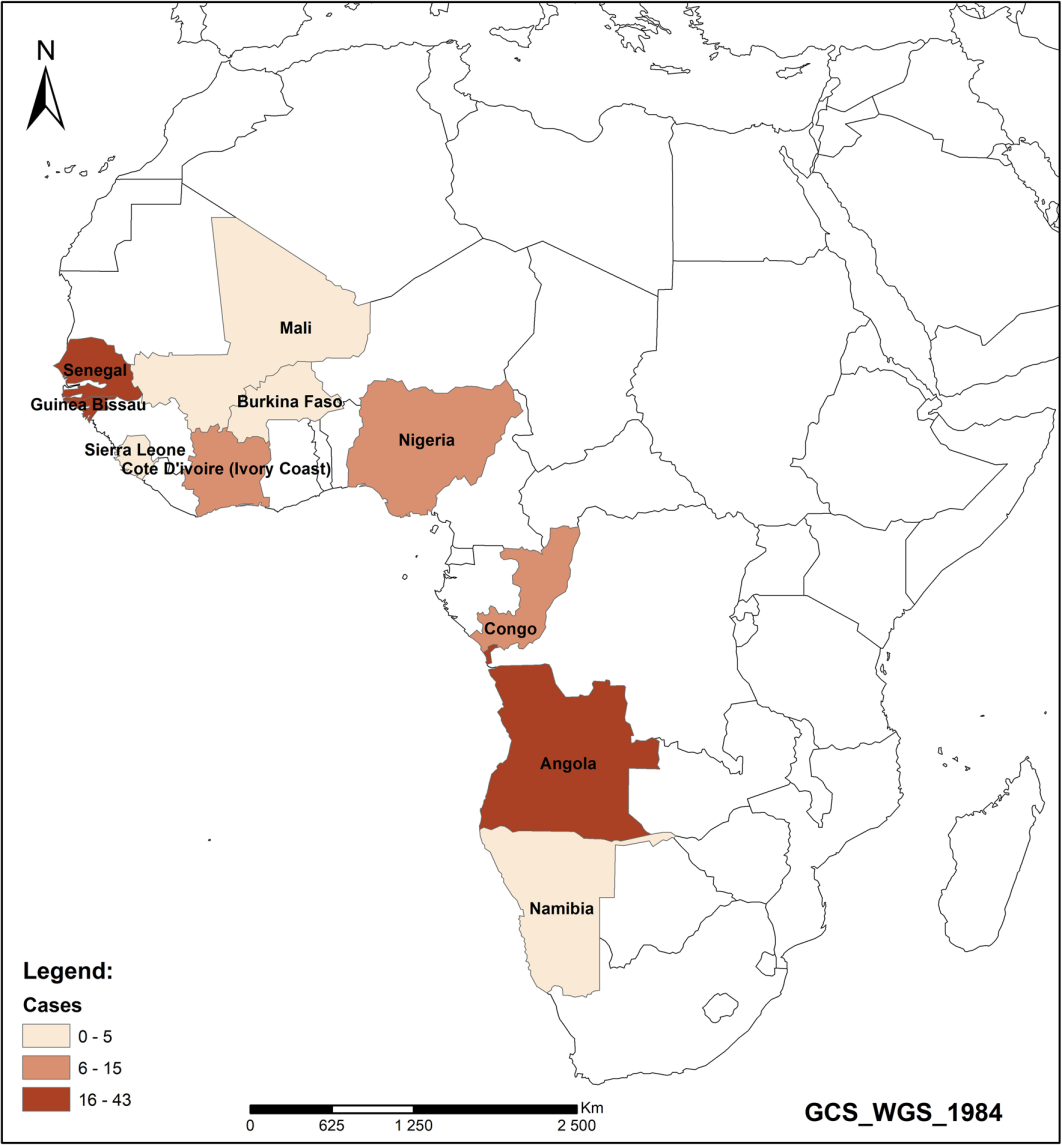
Cumulative number of cases of imported malaria in Cape Verde, 2010–2016

A total of 183 imported malaria cases were recorded from 13 municipalities from Santo Antão, São Vicente, Sal, São Nicolau, Sal, Boavista, Santiago and Fogo during the study period. All the cases originated from different African countries (Fig. 6). Most cases were imported from Angola (46 cases; 25.1%) and Guinea-Bissau (45 cases; 24.6%). Twenty-one cases (11.5%) were from Senegal, and 18 cases (9.8%) were from Equatorial Guinea. Countries such as the Ivory Coast and Nigeria each accounted for 4.4%, with eight imported cases each. Seven imported cases (3.8%) were from Guinea, and the same number originated from the DRC. The remaining imported cases also from the African continent varied between four cases (2.2%) per country to one case per country.

## Discussion

### Malaria situation and the potential for elimination in Cape Verde

Given the epidemiological history of malaria in Cape Verde, some specific time periods with respective profiles can be identified. First, until the 1940s, malaria represented a serious public health problem, with major epidemics, such that the country was considered hyper-endemic, with more than 10,000 cases and more than 200 annual deaths [10]. A second stage, between the years 1967–1972 and 1983–1985, showed that interruption of malaria was possible through the bi-annual IRS campaigns across all the islands. These were the periods when efforts to combat the disease played a significant role in the country. Transmission of malaria was interrupted for 5 years (1968–1972) on the island of Santiago, and then, new cases appeared in Santa Catarina and Santa Cruz (in Santiago), followed by new epidemics in 1973 and 1977–1979. New anti-malarial actions were carried out during 1979–1982, and, consequently, interruption was observed 3 years (1983–1985). A third stage occurred during the last epidemic of 1987, in which the indigenous cases were concentrated mainly on the island of Santiago and occasional cases in Boavista [17]. However, imported cases have been diagnosed in almost the entire national territory, especially the islands most densely populated with the largest number of foreigners, such as São Vicente (20 cases), Sal (11 cases), São Filipe in Fogo (7) and other municipalities in Santiago (especially Praia, with 109 cases, Santa Cruz, Assomada and S. Miguel).

The drastic change in malaria rates in the past 5 years (2010–2016), with an annual incidence below 1%, has led the local Cape Verde authorities to accelerate efforts towards malaria elimination to reach the goal of a malaria-free status by 2020. According to the WHO, a threshold of < 1 case per 1000 inhabitants and 0 malaria-related deaths [4, 18] is required to be eligible for malaria elimination; the country is on track but needs to reinforce its case management and treatment to reduce the disease lethality to zero. Most malaria cases recorded since 2010 were imported from the African continent, which accounts for approximately 90% of the cases recorded worldwide [4]. Imported cases originated mostly from Lusophone countries and the closest neighbouring country (Senegal) that have ongoing endemic malaria transmission; an intense flux of travellers from these countries is facilitated by frequent flight connections. Indeed, in recent years, Praia, the Cape Verde capital city, is connected to Luanda and Bissau, the respective capitals of Angola and Guinea-Bissau, via several airline services. Furthermore, Dakar (Senegal), the nearest capital city, which is approximately 450 km away, can be reached by plane and merchant ferries.

Furthermore, Cape Verde is a tourist country, visited annually by tourists in numbers larger than the resident population. In 2017, approximately 716,000 tourists visited the country, and the goal is to reach 1 million tourists in the coming years. Moreover, foreigners from countries with high endemicity of malaria, including Guinea-Bissau, Nigeria, Guinea, Senegal, Togo and Mali [19], represented 1.6% of the resident population in 2016 (531,239 resident population), with concentrations in certain municipalities, especially in Boavista (12.0%), Sal (5.1%) and Praia (2.4%) [11]. In addition to the ease of mobility of the population to and from the country, mobility between islands is facilitated by several daily flights between islands and frequent sea connections.

Therefore, both infected people and likely vectors frequent reach the archipelago. These data stress the need for reinforced measures to prevent reintroduction of malaria once eliminated owing to the impact of globalization in increasing the risk of introducing imported malaria cases [20]. Moreover, the country will need to be prepared with a strong surveillance system to detect and treat any primary cases early to ensure that they do not become severe, to control the mortality rate, and to monitor all imported cases to prevent malaria resurgence [4].

Indigenous malaria cases in Cape Verde are likely secondary cases infected from primary imported cases. The low annual malaria incidence in the current context of seasonal transmission (July–December) suggests that local transmission is initiated from seasonal migrants returning home from endemic countries, mostly African countries. Transmission is sustained during the rainy season by *An. arabiensis*, the only malaria vector identified in the archipelago [13, 21, 22]. As reported in a recent study, no cases were detected during the dry season in Praia and Boavista, which account for all the indigenous cases in the country [23]. Furthermore, the peak of imported cases corresponds to the peak of vector density, thus supporting the above hypothesis that indigenous cases occur during the rainy season from September to December. However, more epidemiological studies are needed to confirm this hypothesis and better characterize the malaria transmission pattern in Cape Verde.

In the context of consolidating the pre-elimination of malaria, the priority interventions already underway will be strengthened, particularly programme management at the national and local levels. These interventions include vector control, specifically in Santiago (Praia) and Boavista, through larval control and IRS, for more effective control of larval density and longevity of the vector; the management of vector susceptibility of insecticides and the bio-ecological and behavioural monitoring of *An. arabiensis*. Epidemiological surveillance should be strengthened to allow the early detection of cases, the immediate notification and the start of community interventions, in particular, the epidemiological survey (questionnaires and reactive screening) with the reported cases, the focal treatment by IRS around detected cases, and the follow-up of cases after initial treatment. The surveillance of imported cases remains poor in the country, taking into account the existence of four international airports (in Santiago, Sal, São Vicente and Boavista) and the ease of mobility between the islands by air or sea, which thus increase the risk of introducing the disease in the islands without indigenous cases. Therefore, work on communication, awareness and mobilization of malaria elimination with the migrant population and an epidemiological surveillance system at the local level will contribute to the elimination of the disease in the country.

The cost of malaria control in the country is mostly provided by the government of Cape Verde, which aims in its national health policy [17] and other health strategies and policies to eliminate malaria by 2020. The country has strategic and financial partnerships with the Global Fund to Fight AIDS, Tuberculosis and Malaria and the WHO. A number of other partnerships have been developed with other state and private institutions and NGOs. The recent implementation of the Laboratory of Medical Entomology of the National Institute of Public Health and the various collaborations with universities and research institutions at the national and international level will allow for better research on malaria in the country, thus enabling the best strategies to control and eliminate the disease.

### Strong malaria management system

The country has made significant progress towards better management of malaria and improvement of the national health system. According to the WHO framework for malaria elimination, countries need to transform their malaria surveillance into a core intervention and strengthen the health structure to ensure universal access to malaria prevention, diagnosis and treatment [4]. The government of Cape Verde aligned its renewed malaria strategic plan [17] with WHO recommendations to better organize the national health structure to respond adequately to all cases of malaria diagnosed in the country, thus reducing the disease incidence to less than 1 annual case per 1000 inhabitants as well as the malaria-related mortality. The country has improved health structure coverage, with two central hospitals, four regional hospitals, 31 health centres, 17 health delegations, five reproductive health centres, 34 health posts and 107 health units, ensuring that any inhabitant is less than 5 km from a health institution. In all health organizations, health care is provided by trained technicians (doctors, nurses and laboratory technicians) to efficiently detect and treat any malaria case that occurs anywhere in the archipelago. All malaria cases confirmed by RDT and microscopy should then treated be according to the national guideline of treatment. The diagnostic coverage is universal and free of charge in all public health structures. Microscopy is available in 100% of health structures with laboratory capacity, RDT are present in all health structures, and the technicians are properly trained for clinical and laboratory diagnosis, with frequent recycling sessions [24]. The supply chain is in place in the country for the management of cases and to ensure that supplies remain in stock.

Despite all the efforts to strengthen the national malaria management system, malaria-related deaths still occur in the country. One reason for this is the lack of patient knowledge about malaria and attitudes towards health care, especially in the imported cases. According to the 2016 epidemiological data, the average time between the first symptoms of the disease and the patient visit to the nearest health services was on average 3 days (ranging from 0 to 9 days). For indigenous cases, the average delay is 2.6 days, and the delay greater for imported cases, with a mean of 4.2 days. However, in Praia (the most affected community), the average time between symptoms and the visit to a health service was 2.1 days in 2016 [25]. This difference is even greater according to the patient origin of the cases. Patients with imported cases from countries with high endemicity take more time to seek care than patients from Praia.

In this situation, one of the corrective measures is to reinforce behavioural change in collaboration with the media and field agents to enhance awareness about malaria and reduce the lag-time between the first symptoms and a healthcare visit. This will ultimately prevent deaths from malaria in the country and allow the country to achieve its elimination goal.

### Malaria in Cape Verde archipelago *vs* other African regions

In contrast to others African regions where most of malaria cases are primarily found in the high-risk groups of children under 5 years of age and pregnant women [3], in Cape Verde, the annual number of malaria cases in children < 5 was only 3% during the whole study period. In the meantime, no cases were recorded among pregnant women. In the archipelago, most patients were young adult men of 20 years old, especially those spending time outside for fun or due to their professional activities, such as security agents, or their social condition (homeless and other social classes). These groups have increased exposure to infected mosquito bites since *An. arabiensis* is known for its marked exophagic and exophilic behaviour [26].

Notably, during the last few years, all indigenous cases in Cape Verde were recorded in only two of the nine inhabited islands, namely, Santiago island, with its capital city, Praia, which accounts for the majority of cases, and Boavista island, where 45 cases occurred. Both islands are the two most visited places of the archipelago with high migrant fluxes from several African countries, especially from malaria-endemic Lusophone countries. These migrants are the main source of imported malaria cases from which the indigenous cases originate.

In general, the majority of the population in Cape Verde is considered to be at risk of malaria among non-immune groups in an area prone to malaria outbreaks. The reasons for this are as follows: (i) 58% of the total population lives on islands with local malaria transmission in the Santiago and Boavista islands, and (ii) although local transmission was not recorded until now on other islands, the presence of the vector constitutes a risk factor for approximately 37% of the non-immune population. Only 5% of the local population is living in a non-malarial area without the vector. Moreover, the non-immune status of the archipelago population with the important internal and external migratory fluxes, especially the movement of infected people from neighbouring malaria-endemic countries, are among other factors rendering the local population at risk of malaria transmission and the area prone to malaria outbreaks [17].

## Conclusions

Epidemiologically speaking, malaria in Cape Verde is unstable, with sporadic seasonal transmission, is highly variable from year to year, and has a peak of transmission during the rainy season. Most of the archipelago malaria cases were imported and recorded on the nine inhabited islands, with an important inter-annual variation. Indigenous cases occurred only in the two most visited islands, especially Santiago island, where almost all cases were recorded in Praia, the capital city. Most potentially infected migrants visit and/or transit through Praia. Twenty-year-old men were the group at the highest risk, while children under five were less impacted, and no infection in a pregnant woman has been recorded yet.

Cape Verde is still on track to achieve its malaria elimination goal by 2020 due to the reduction of the annual incidence to below 1%. However, more needs to be done to reduce malaria lethality to zero. The archipelago of Cape Verde is a good example of the government’s ability to tackle malaria and work toward its elimination by strengthening the health as well as surveillance systems.
